# Comparative transcriptome analysis reveals key long noncoding RNAs for cadmium tolerance in Tibetan hull-less barley

**DOI:** 10.3389/fpls.2025.1572490

**Published:** 2025-05-22

**Authors:** Md Rafat Al Foysal, Cheng-Wei Qiu, Feibo Wu

**Affiliations:** ^1^ Department of Agronomy, College of Agriculture and Biotechnology, Zhejiang University, Hangzhou, China; ^2^ Department of Agronomy and Haor Agriculture, Faculty of Agriculture, Sylhet Agricultural University, Sylhet, Bangladesh

**Keywords:** Hordeum vulgare var. nudum, Cd toxicity, high-throughput sequencing, lncRNA, mRNA, target genes

## Abstract

Cadmium (Cd) is one of the most hazardous and persistent heavy metal pollutants globally. Long noncoding RNAs (lncRNAs) play a crucial role in regulating plant gene expression under various abiotic stress conditions. This study investigated the response of the lncRNA transcriptome in the roots of two contrasting Tibetan hull-less barley genotypes, X178 (Cd-tolerant) and X38 (Cd-sensitive), to Cd stress using RNA sequencing. A total of 8299 novel lncRNAs were identified, with 5166 unique target genes associated with 2571 unique lncRNAs. Among these, 1884 target genes were regulated by cis-acting lncRNAs, while 3428 were regulated by trans-acting lncRNAs. By analyzing differential expression profiles in the two genotypes under Cd stress, 26 lncRNAs and 150 mRNAs were identified as potentially linked to Cd tolerance. Functional enrichment analysis revealed that the target genes were significantly enriched in detoxification and stress response functions, including pathways related to phenylalanine, tyrosine, tryptophan, ABC transporters, and secondary metabolites. Additionally, 12 lncRNAs forming 18 lncRNA-mRNA pairs were identified as key regulators of Cd tolerance. The functional roles of these lncRNA-mRNA interactions suggest that they modulate proteins such as DJ-1, EDR, PHT, and ABC transporters, which may contribute to the Cd tolerance observed in genotype X178. High-throughput sequencing results were validated by qRT-PCR. These findings deepen our understanding of lncRNAs as critical regulators of Cd tolerance in plants, offering valuable insights into the molecular mechanisms underlying heavy metal stress responses in crops.

## 1 Introduction

Heavy metal contamination of agricultural soils, particularly cadmium (Cd), poses significant threats to plant life, human health, and global food security. Cd is one of the most hazardous and persistent toxic heavy metals in the environment, known for its high toxicity and ease of uptake by plants (Zhou et al., 2023; Angon et al., 2024). Soil Cd contamination not only adversely affects plant growth, photosynthesis, and reduces yield and quality, but also signifies a serious health risk to humans through the food chain (Genchi et al., 2020; Kaur et al., 2021). The detrimental effects of heavy metal contamination are particularly pronounced in agricultural fields located near industrial or mining activities, which are major sources of such pollutants (Ullah et al., 2024, Ullah et al, 2025). Global anthropogenic Cd fluxes now approach 1.0 × 10^6^ metric tons annually (Ren et al., 2022), with the topsoils of the European Union exhibiting an average concentration of 0.20 mg kg^–1^ (Ballabio et al., 2024), reflecting its persistent environmental mobility and importance in biogeochemical cycling. Cd has been identified as the leading contributor to carcinogenic risk among heavy metals. According to guidelines from the US Environmental Protection Agency (USEPA), the acceptable threshold for regulatory consideration of cancer risk ranges from 1×10^–6^ to 1×10^–4^ (Kamunda et al., 2016). Notably, the USEPA has classified Cd as a priority hazardous metal, ranking it 118th on the Priority Pollutant List—a ranking higher than other toxic heavy metals, including chromium (Cr), copper (Cu), and lead (Pb).

To ensure sustainable agricultural productivity and ecosystem health, it is crucial to exploit available genetic resources and better understand the mechanisms of Cd tolerance. This understanding is essential for developing cultivars with low Cd accumulation and high tolerance, thus reducing Cd intake in human diets and minimizing soil-plant Cd transfer. In response to Cd contamination, plants have evolved a variety of adaptive strategies and detoxification mechanisms (Qiu et al., 2021). These include Cd exclusion, chelation by specific ligands, sequestration in vacuoles, and the activation of antioxidative defense systems to neutralize excess reactive oxygen species (ROS) (Luo and Zhang, 2021; Wang et al., 2023).

Long non-coding RNAs (lncRNAs) have emerged as pivotal regulators of gene expression at both transcriptional and post-transcriptional levels, playing essential roles in processes such as hormone signaling, plant development, and responses to abiotic stresses (Li et al., 2018; Nejat and Mantri, 2018; Wang et al., 2018; Moison et al., 2021);. A growing body of research has linked numerous lncRNAs to Cd stress responses across various plant species. For instance, 195 Cd-responsive lncRNAs were identified in barley (Zhou et al., 2023), 301 in *Brassica napus* (Feng et al., 2016b), and 172 in *Populus tomentosa* (Quan et al., 2021). In rice, Chen et al. (2018) reported that Cd treatment resulted in the downregulation of 75 lncRNAs and the upregulation of 69 lncRNAs. Among these, XLOC_086307, which targets the Cys-rich peptide metabolism-related gene Os03g0196600 in cis, was significantly upregulated, suggesting its involvement in the Cd response. Additionally, the TCONS_00035787–miR167-Nramp1 pathway highlights the role of lncRNAs in modulating Cd uptake and accumulation (Feng et al., 2016b). In *Betula platyphylla*, LncRNA2705.1 and LncRNA11415.1 have been shown to enhance Cd tolerance by regulating the expression of target genes such as *LDHA* and *HSP18.1* (Wen et al., 2020). Despite these advances, the regulatory mechanisms of lncRNAs in Tibetan hull-less barley under Cd stress remain largely unexplored, especially considering its unique adaptation to extreme environmental conditions—a critical gap given its ecological and agricultural importance.

Furthermore, transcriptome screening identified 9414 lncRNAs associated with seed coat coloration and anthocyanin biosynthesis in Tibetan hull-less barley, providing a dynamic portrait of these processes (Zheng K, et al., 2023). Additionally, 14 mowing-responsive lncRNAs were found to interact with target genes involved in cytokinin signaling, cell wall degradation, storage protein accumulation, and biomass increase, playing a critical role in the regeneration of hull-less barley after mowing (Bai et al., 2024). However, no study has yet linked lncRNAs to Cd tolerance in Tibetan hull-less barley, nor elucidated whether its lncRNA-mediated regulatory networks differ from other barley species under Cd exposure. This knowledge gap necessitates further investigation.

Tibetan hull-less barley (*Hordeum vulgare* var. *nudum*) is a vital staple food and economic crop for populations in Tibet and surrounding regions. Cultivated under the harsh conditions of the Qinghai-Tibet Plateau, it endures extreme altitudes and environmental stresses. Remarkably, Tibetan hull-less barley has developed exceptional tolerance to these challenges, surpassing other barley species in its resilience. In our previous study, we identified two distinct genotypes of Tibetan hull-less barley: X178, which is tolerant to Cd, and X38, which is Cd-sensitive (Foysal et al., 2024). This discovery raises the question of whether Tibetan hull-less barley employs a unique Cd-tolerance strategy at the transcriptome level, particularly through the regulation of lncRNAs that may contribute to its environmental adaptability—a hypothesis yet to be tested.

We hypothesize that genotype-specific lncRNA networks that differentially control significant genes are responsible for the difference in Cd tolerance between Tibetan hull-less barley genotypes X178 and X38. The identification, functional roles, regulatory mechanisms, and crop enhancement applications of lncRNAs in Tibetan hull-less barley are poorly understood. To address this, the present study investigates the molecular mechanisms underlying the root lncRNA transcriptome in response to Cd stress using RNA sequencing under both Cd stress and normal conditions. This research offers valuable insights that could contribute to the development of Cd-tolerant barley cultivars, enhancing crop resilience in challenging environments.

## 2 Materials and methods

### 2.1 Plant materials and Cd treatment

A greenhouse hydroponic experiment was conducted at the Zhejiang University, Zijingang Campus in Hangzhou, China. Healthy barley seeds of two genotypes, Tibetan hull-less barley X178 (Cd-tolerant) and X38 (Cd-sensitive), were germinated in a plant growth chamber (22/18°C day/night, relative humidity 65% and light intensity 250 µmol m^–2^ s^–1^) on sterilized filter paper in Petri dishes. Germination took place over 3 days in the dark, followed by 2 days under light conditions. Five-day-old, uniform, and healthy seedlings were transferred into 5 L plastic containers, each containing 4.8 L of a basic nutrient solution (BNS) prepared according to Qiu et al. (2019), and continued to grow in the chamber under identical temperature, humidity, and light settings. After 5 days, the control group received BNS, while the Cd-treated group was provided with BNS supplemented with 20 µmol L^-1^ CdCl_2_. This concentration was selected based on previous research, which showed it induces significant phenotypic differences between the tolerant and sensitive genotypes (Foysal et al., 2024). It effectively facilitates the investigation of underlying transcriptional regulatory mechanisms and has environmental significance concerning physiological responses in barley (Chen et al., 2008; Schneider et al., 2009). A split-plot design was used, with the treatment as the main plot and genotype as the subplot, and three biological replicates for each treatment. After 24 hours of Cd exposure, root samples were collected from each replicate, frozen in liquid nitrogen, and stored at -80°C for RNA extraction. Young seedlings exhibit heightened sensitivity to environmental stress, with a 24-hour treatment effectively inducing stress responses to identify key regulatory genes/lncRNAs in early stress adaptation (Wen et al., 2020). This developmental phase coincides with critical root architecture formation through active cell division and differentiation, making it optimal for studying dynamic gene expression patterns (Duan et al., 2023; Liang et al., 2024).

### 2.2 Preparing transcriptome libraries and high-throughput sequencing

RNA isolation, purification, library construction, and high-throughput sequencing were performed as described by Qiu et al. (2019). In brief, total RNA was first subjected to ribosomal RNA (rRNA) removal using RNase H. The purified RNA was then fragmented, and first-strand cDNA was synthesized using random hexamer primers for reverse transcription. This was followed by second-strand cDNA synthesis with dUTP replacing dTTP. The resulting double-stranded cDNA underwent end repair, “A” base addition, and adaptor ligation. RNA sequencing libraries were generated by PCR amplification, followed by digestion of the U-labeled second-strand template using Uracil-DNA glycosylase (UDG). Library quality was assessed, and the PCR product was denatured by heat. The DNA was then circularized using splint oligo and DNA ligase. DNA nanoball (DNB) synthesis and sequencing were carried out on the DNBSEQ platform (BGI-Wuhan).

### 2.3 Mapping and classification of transcripts

Clean data were obtained by removing reads containing adapter sequences, poly-N regions, and other low-quality reads from the raw data. Low-quality reads were filtered based on the criterion that more than 50% of the bases in a read must have a sequencing quality of no more than 15. The clean reads were then mapped to the barley reference genome (Barley_Morex_V2) using HISAT2 (Version: v2.2.1; Parameters: –sensitive –no-discordant –no-mixed -I 1 -X 1000 -p 8 –rna-strandness RF; https://github.com/DaehwanKimLab/hisat2/) and assembled with StringTie (Version: v2.2.1; Parameters: -f 0.3 -j 3 -c 5 -g 100 -s 10000 -p 8; https://github.com/gpertea/stringtie/). Known mRNAs and lncRNAs were identified using Cuffcompare (https://github.com/gpertea/CuffCompare/). To identify novel transcripts, we used CPC (http://cpc2.cbi.pku.edu.cn/), LGC (https://ngdc.cncb.ac.cn/lgc/calculator/), CNCI (https://github.com/www-bioinfo-org/CNCI/), Pfam (http://pfam-legacy.xfam.org/), and SwissProt (https://www.uniprot.org/) for coding ability prediction. The thresholds for each tool were as follows: for CPC and CNCI, transcripts with a score greater than 0 were classified as mRNAs, while those with a score less than 0 were classified as lncRNAs. For LGC and CNCI, a score threshold of 0 was also applied, with transcripts greater than 0 classified as mRNAs and those less than 0 classified as lncRNAs. Pfam and SwissProt are protein databases, and transcripts that could be aligned with these databases were considered mRNAs, while those that could not were classified as lncRNAs. A transcript was classified as mRNA if at least three of the five methods (CPC, LGC, CNCI, Pfam, or SwissProt) were consistent, and at least one protein database alignment was detected.

### 2.4 Analysis of mRNA and lncRNA expression

The software Bowtie2 (https://github.com/BenLangmead/bowtie2/) was used to align the clean reads to the reference sequence, followed by RSEM (https://github.com/deweylab/RSEM/) to calculate gene and transcript expression using the FPKM (Fragments Per Kilobase of transcript per Million mapped reads) method. Differential expression analysis of transcripts between control and Cd treatment groups for each genotype was performed using DEGseq (https://github.com/bioc/DEGseq/). Transcripts with a false discovery rate (FDR) ≤ 0.05 and |log_2_ fold change| ≥ 1 were considered differentially expressed mRNAs or lncRNAs.

### 2.5 Real-time quantitative PCR validation

The qRT-PCR was employed to validate the high-throughput sequencing results of barley lncRNAs and mRNAs by analyzing the expression levels of 6 lncRNAs and 4 mRNAs. RNA samples were reverse-transcribed using the PrimeScript™ II 1st Strand cDNA Synthesis Kit (Takara). The qRT-PCR reaction conditions were as follows: initial denaturation at 95°C for 30 seconds, followed by 40 cycles of 95°C for 3 seconds and 60°C for 30 seconds. The specificity of PCR amplification was confirmed through melting curve analysis. All reactions were performed in triplicate using the SYBR Premix Ex Taq Kit (Takara) on a Light Cycler 480 System (Roche, Germany), following the manufacturer’s instructions. Amplification efficiency was validated by analyzing the linear regression of log-transformed template quantities versus Ct values during the exponential phase of amplification curves. Relative expression was calculated using the comparative Ct (2^−ΔΔCt^) method, normalizing target gene Ct values to a reference gene and control group, with the *GAPDH* gene serving as the reference gene (Qiu et al., 2019). Primer sequences used in this study are provided in **Supplementary Table S1**.

### 2.6 Target and sRNA precursor prediction

LncRNAs primarily exert their functions through cis- or trans-regulation of target genes. To investigate this, we computed two correlation coefficients (Spearman and Pearson) between lncRNAs and mRNAs, requiring both coefficients to exceed 0.6. LncRNAs were classified as cis-regulatory if located within 10 kb upstream or downstream of coding genes. For lncRNAs outside this range, we used RNAplex to analyze the binding energy between lncRNAs and mRNAs, considering an energy threshold of -60 kcal mol^-1^ or lower to define trans-regulatory interactions. Additionally, we employed BLAST to map lncRNAs to miRBase (http://www.mirbase.org/) to identify potential small RNA (sRNA) precursors, as lncRNAs can also function as precursors for sRNAs.

### 2.7 mRNA and lncRNA annotation

BLAST was performed to align mRNA sequences to the NT database, and DIAMOND (https://github.com/bbuchfink/diamond/) was used to align translated sequences to the NR, KOG, KEGG, Uniprot, COG, and TFdb5 databases. Additionally, INFERNAL (https://github.com/EddyRivasLab/infernal/) was employed to map lncRNAs to the Rfam database for annotation of the lncRNA family. Gene Ontology (GO) annotation for target genes of differentially expressed lncRNAs was conducted using the GOseq R package (https://github.com/lmika/goseq/). KEGG pathway enrichment analysis of target genes for differentially expressed lncRNAs was performed using KOBAS (https://github.com/KOBAS-Development/KOBAS/) software.

## 3 Results

### 3.1 Identification of lncRNAs in Tibetan hull-less barley under control and Cd stress

**Figure 1:**
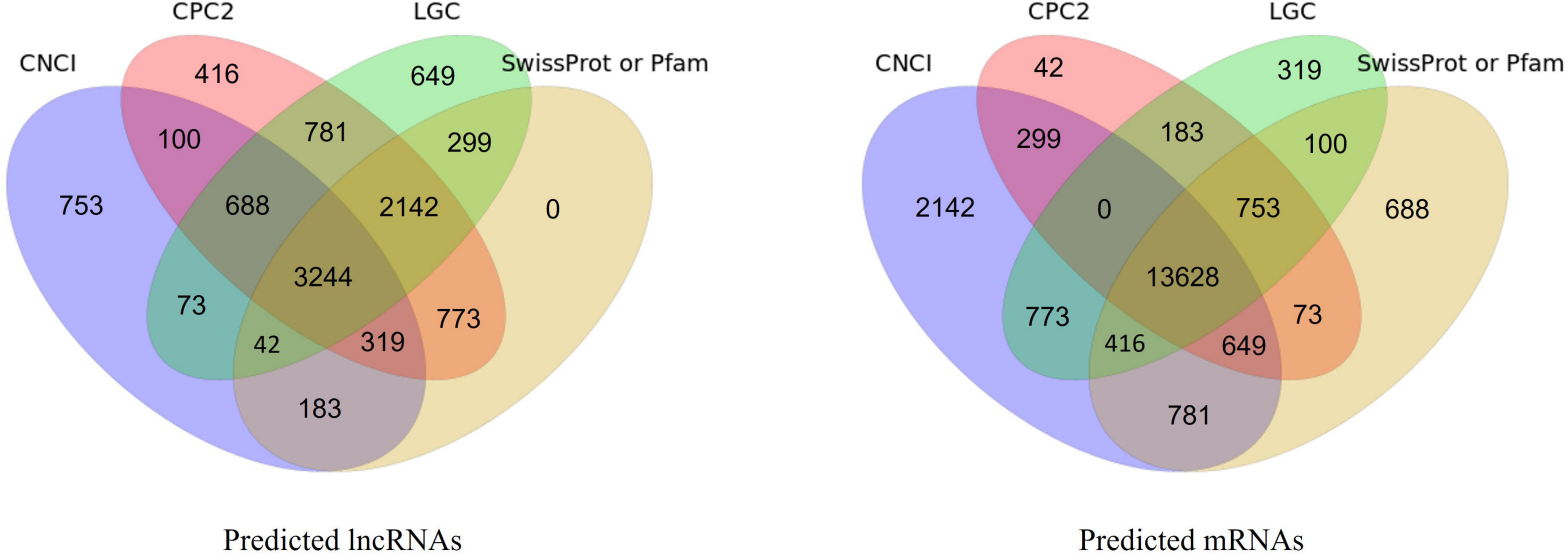
Coding potential prediction of lncRNAs and mRNAs by CNCI, CPC2, LGC, and SwissProt/Pfam. Venn diagrams show number of predicted lncRNAs (left) and mRNAs (right).

**Figure 2:**
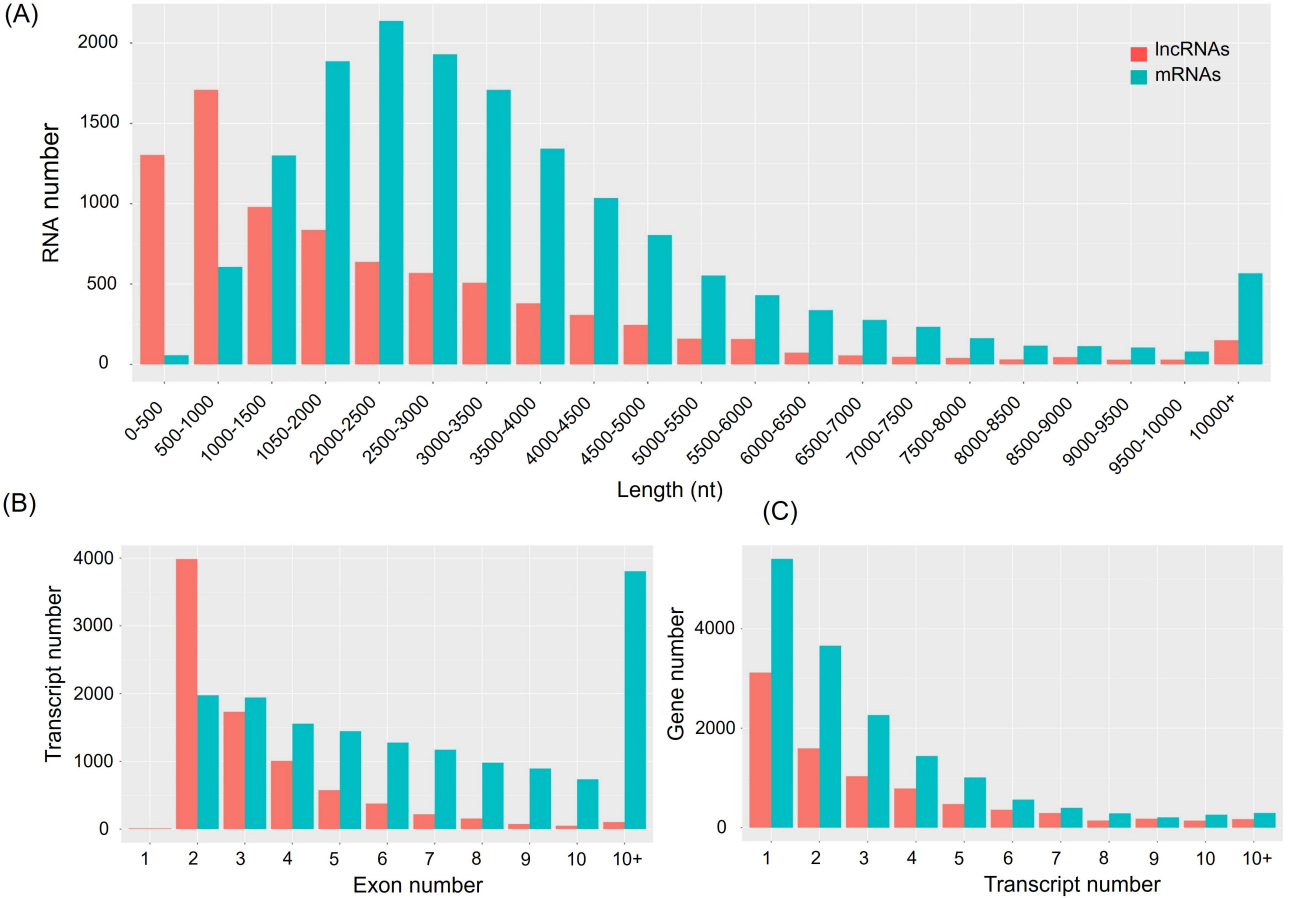
Characterization of all lncRNAs and mRNAs. **(A)** Length distribution of lncRNAs and mRNAs. **(B)** The exon features of lncRNAs and mRNAs. **(C)** The transcripts number of genes and lncRNA loci.

Morphological changes induced by Cd treatment are presented in **Supplementary Figure S1**. Physiological evaluations revealed that, under Cd stress, growth parameters such as shoot height, root length, fresh weight, and dry weight were significantly higher in the X178 variety compared to X38 (**Supplementary Figure S2**). The Illumina HiSeq platform was employed for RNA-sequencing, generating 56.46 and 56.48 million raw reads for the X178 and X38 control libraries, respectively. Similarly, 56.45 and 55.64 million raw reads were obtained for the Cd stress libraries (**Table 1**). Following the removal of low-quality reads, a total of 55.22, 55.20, 55.27, and 54.36 million high-quality clean reads were retained for the X178-Control, X38-Control, X178-Cd, and X38-Cd libraries, respectively. Using the HISAT alignment tool, approximately 83% of the clean reads were successfully mapped to the reference genome (**Table 1**). To identify putative lncRNAs in Tibetan hull-less barley, transcripts shorter than 200 nucleotides (nt) and known mRNAs were excluded. The coding potential of the remaining transcripts was assessed using four computational tools: CNCI, CPC2, LGC, and SwissProt or Pfam databases (**Figure 1**). Through this comprehensive analysis, we identified 8299 novel lncRNAs, 15791 novel mRNAs, and 63658 known mRNAs (**Figure 2**). The majority of lncRNAs were under 1000 nt, with fewer found as their length increased. Most lncRNAs were under 2000 nt in length, whereas mRNAs predominantly ranged between 500 and 3500 nt. Additionally, lncRNAs were predominantly characterized by two exons, while mRNAs exhibited a significantly higher proportion of transcripts with more than two exons. The transcript quantity distribution followed a similar trend. These findings suggest distinct structural and functional roles for lncRNAs and mRNAs in biological processes, highlighting their differential contributions to gene regulation and cellular functions.

**Table 1:** Summary of high-throughput sequencing of transcripts from barley roots.

Library	Control		Cd	
	X178	X38	X178	X38
Total raw reads (Millions)	56.46	56.48	56.45	55.64
Total clean reads (Millions)	55.22	55.20	55.27	54.36
Total clean reads ratio (%)	97.79	97.73	97.90	97.71
Total Mapping Ratio (%)	86.08	77.27	86.26	81.34
Uniquely mapping ratio (%)	77.44	70.64	78.82	74.68
Novel lncRNA gene	2149	2140	2145	2097
Novel lncRNA isoforms	6957	6944	6948	6804
Novel mRNA gene	158	161	162	157
Novel mRNA isoforms	14050	13976	14071	13963
Known mRNA gene	31809	31870	31696	31622
Known mRNA isoforms	28415	28739	28356	28376

Control and Cd, the plants were treated with basic nutrient solution (BNS) and BNS + 20 µmol L^-1^ Cd, respectively.

### 3.2 lncRNAs family classification and target gene prediction

The lncRNA families were classified based on their secondary structures and conserved sequences (**Supplementary Table S2**). A total of 2234 lncRNAs were categorized into distinct families, with the majority distributed among several key families. Specifically, 22.24% of the lncRNAs were classified under the MIR1122 family (id: RF00906), followed by 17.19% in the P6 family (id: RF01683), 12.67% in the mir family (id: RF00756), 7.97% in the RUF21 family (id: RF01825), 7.29% in the IRES_c family (id: RF00216), 5.82% in the RNase_P family (id: RF01577), 3.94% in the MIR1023 family (id: RF01043), and 3.94% in the SSU_rRNA_eukarya family (id: RF01960). The remaining 235 families, with an average of 6.0 individuals per family, collectively accounted for 18.93% of the total lncRNAs analyzed. These results demonstrate that lncRNAs exhibit significant structural diversity and are closely associated with other non-coding RNA families, underscoring their potential importance in biological functions and regulatory mechanisms.

LncRNA-mediated gene expression regulation can occur through either cis or trans mechanisms. Here, 5166 unique target genes were identified for 2571 unique lncRNAs, accounting for 31.0% of the total lncRNAs identified. The mechanisms of action for many lncRNAs remain unknown, underscoring the complexity of lncRNA functions in biological processes. Among the target genes, 1884 were found to be regulated by cis-acting lncRNAs, while 3428 were regulated by trans-acting lncRNAs (**Supplementary Table S3**, **S4**). Of the 3359 lncRNA-mRNA pairs involved in cis-regulation, 893 target genes were located within 10 kilobases (kb) downstream of lncRNA loci, 664 were positioned within 10 kb upstream of lncRNA loci, and 1802 were found to overlap with lncRNA loci (**Supplementary Table S3**). The overlapping interactions were further classified into specific subtypes, including AntiIntronic (57), AntiOverlapping (21), AntiSense (173), Intronic (118), Overlapping (53), and Sense (1380).

### 3.3 Identification of lncRNAs as sRNA precursors

LncRNAs have been identified as potential precursors for microRNAs (miRNAs), which play a role in gene regulation. Using BLAST, lncRNAs were mapped to the miRBase database to identify potential miRNA precursors. A total of 25 lncRNAs were found to potentially serve as precursors for miRNAs in the selected species. This suggests that these lncRNAs may generate miRNAs in response to Cd stress. Notably, several of these lncRNAs were predicted to produce barley miRNAs, including hvu-MIR5049c, hvu-MIR6200, hvu-MIR5049a, hvu-MIR5049e, hvu-MIR159a, hvu-MIR6190, hvu-MIR156b, hvu-MIR156a, hvu-MIR166a, hvu-MIR166c, hvu-MIR444a, hvu-MIR5048b, hvu-MIR5048a, and hvu-MIR6205 (**Supplementary Table S5**). These findings highlight the potential role of lncRNAs in miRNA biogenesis and their involvement in stress-responsive regulatory mechanisms.

### 3.4 Differences in lncRNA and mRNA expression profile among two barley genotypes

**Figure 3:**
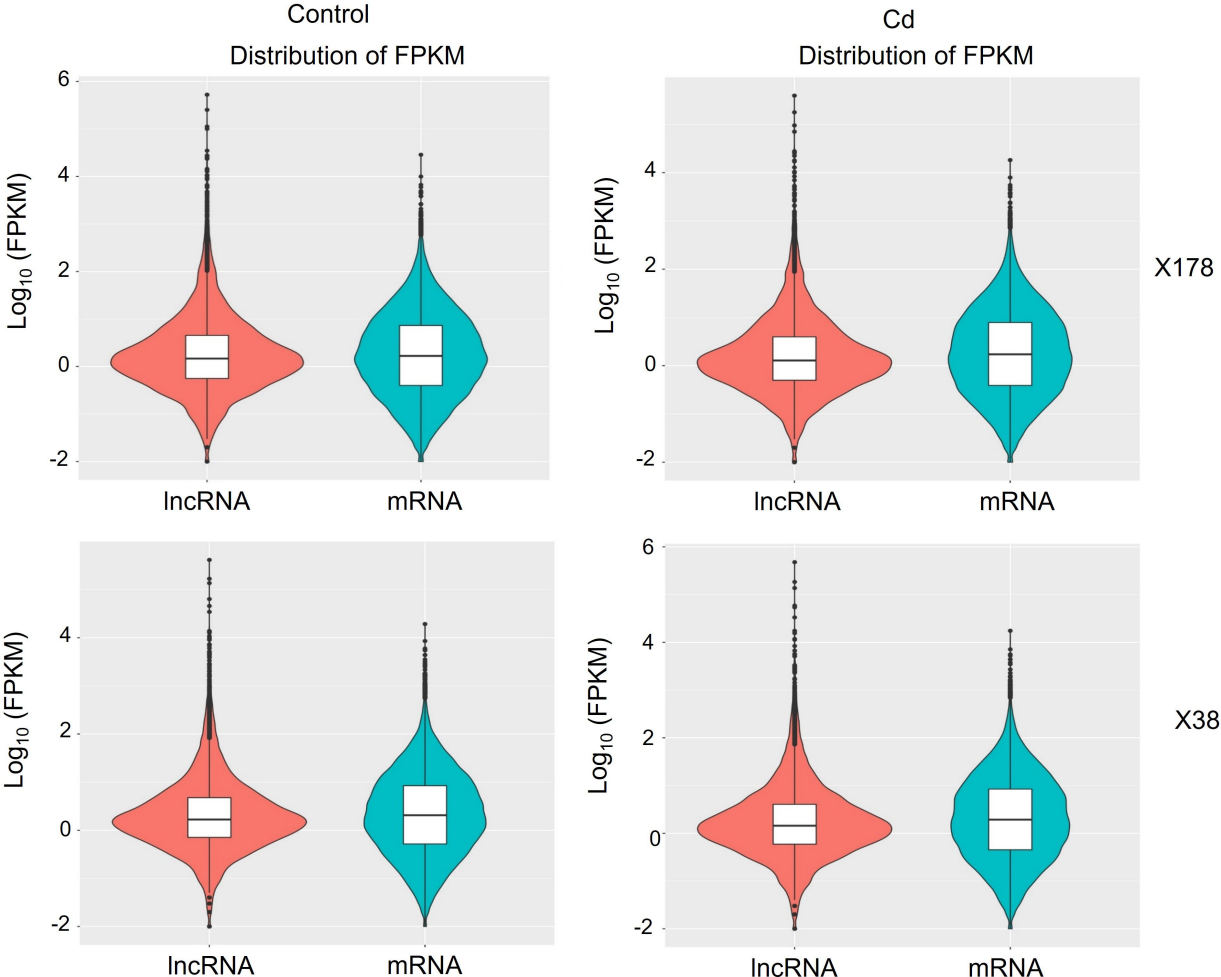
Expression levels and densities of mRNA and lncRNA in Cd-tolerant (X178) and Cd-sensitive (X38) barley genotypes under 20 µmol L^-1^ Cd stress. X-axis: RNA density; Y-axis: log_10_(FPKM). Colors denote RNA type. Box plot: median (black line), quartiles (box), and data range (whiskers).

To identify Cd-responsive lncRNAs and mRNAs, the expression levels of these transcripts were compared between control and Cd stress conditions. Differential expression patterns were observed between the Cd-treated and control groups, as indicated by the subtle shape differences in the expression profiles (**Figure 3**). Specifically, in X178, 133 lncRNAs and 1587 mRNAs were up-regulated, whereas 160 lncRNAs and 932 mRNAs were down-regulated (**Supplementary Table S6**). Similarly, in X38, 51 lncRNAs and 717 mRNAs were up-regulated, while 148 lncRNAs and 407 mRNAs were down-regulated (**Supplementary Table S7**). These results highlight the distinct transcriptional responses of lncRNAs and mRNAs to Cd stress in the two genotypes. Up-regulated transcripts were linked to detoxification pathways (e.g., glutathione metabolism, phytochelatin synthesis, ABC transporters) and stress signaling (MAPK, antioxidant systems), potentially enhancing Cd tolerance. Down-regulated transcripts correlated with suppressed growth processes (e.g., photosynthesis), suggesting metabolic reallocation for stress defense. Co-regulated lncRNAs may modulate these responses by interacting with transcription factors or mRNA networks, while their suppression could release inhibitory constraints. This interplay between coding and non-coding RNAs underscores their synergistic roles in balancing detoxification, stress adaptation, and metabolic homeostasis under Cd stress.

**Figure 4:**
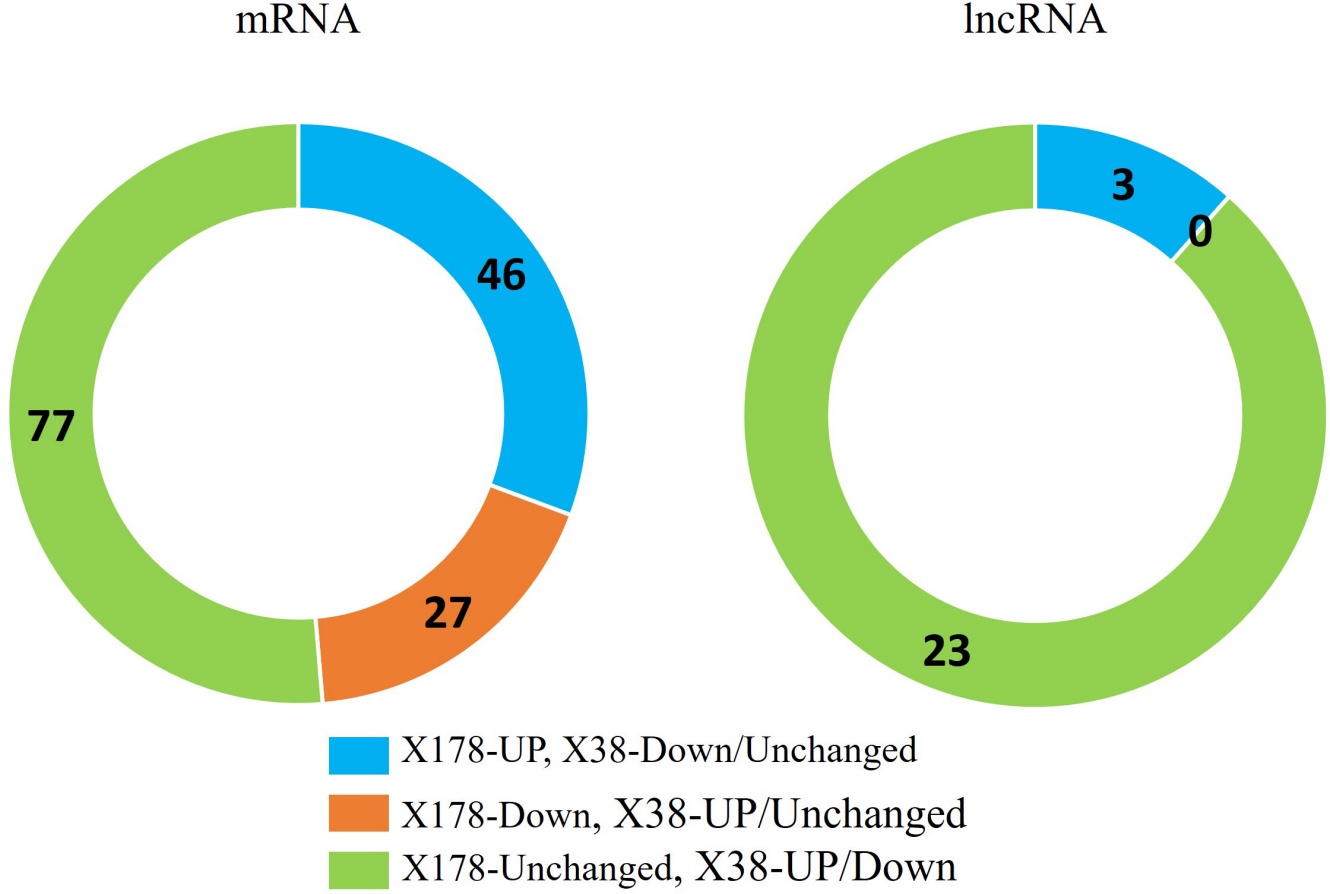
Root transcriptome profiles of Cd stress-responsive mRNAs and lncRNA in Cd-tolerant (X178) and Cd-sensitive (X38) barley genotypes under 20 µmol L^-1^ Cd stress. log_2_N, log_2_N ≥ 1 are up-regulated, between 0 < |log_2_N| < 1 are unchanged and log_2_N ≤ − 1 are down-regulated.

**Figure 5:**
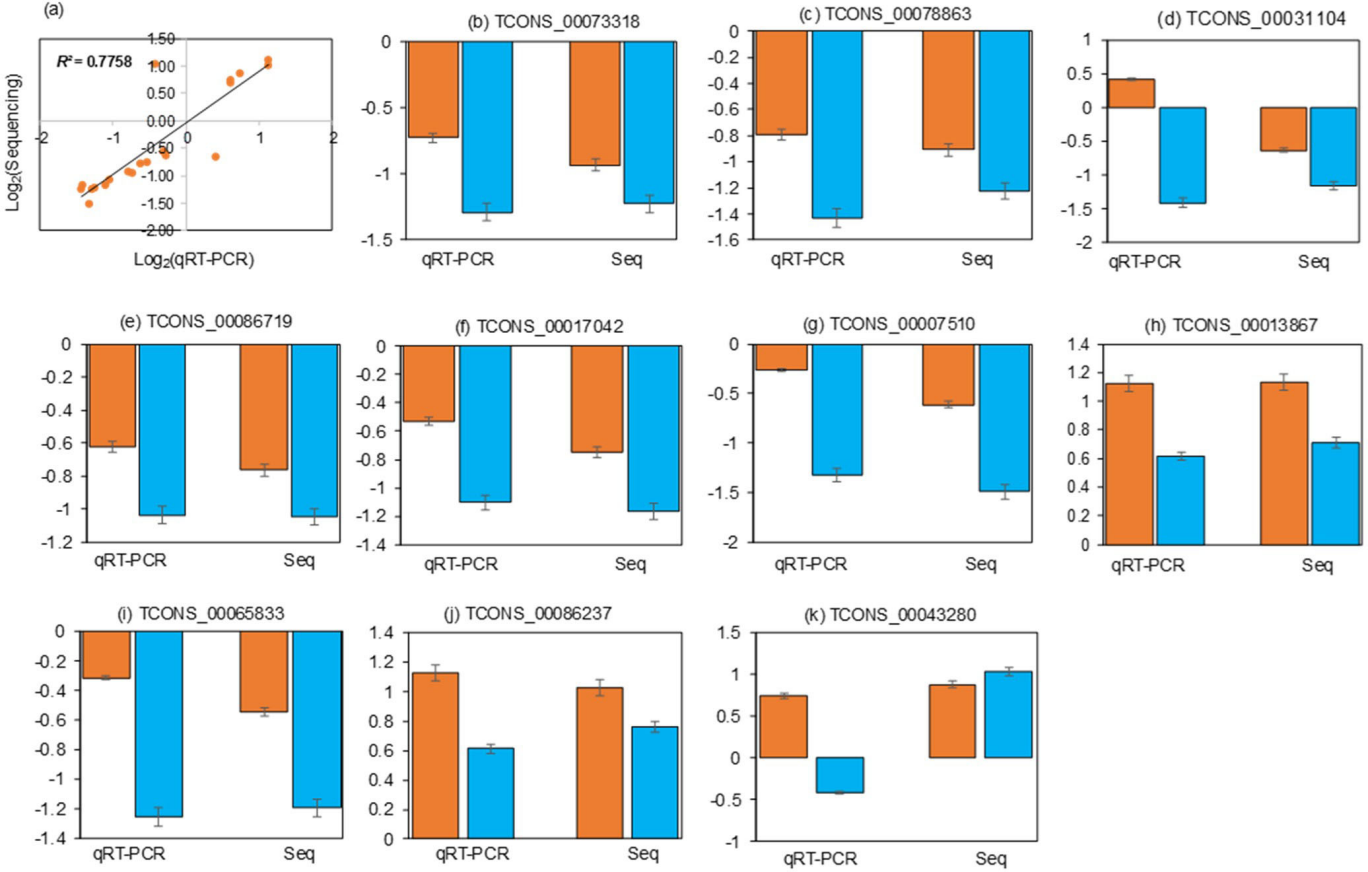
Correlation between lncRNA/mRNA sequencing and qRT-PCR data. **(a)** Fold changes from sequencing (Y-axis) versus qRT-PCR (X-axis) for X178 and X38 under Cd stress. R² indicates linear regression fit quality. **(b-k)** Validation of six lncRNAs and four mRNAs by qRT-PCR in X178 (orange) and X38 (blue) under Cd stress. “Seq” denotes high-throughput sequencing data.

To identify lncRNAs and mRNAs associated with Cd tolerance, the Cd-responsive lncRNAs and mRNAs were compared between the X178 and X38. A total of 26 lncRNAs and 150 mRNAs were identified as potentially linked to Cd tolerance (**Figure 4**). Among these, 3 lncRNAs were up-regulated in X178 but either down-regulated or unchanged in X38, while 23 lncRNAs remained unchanged in X178 but were either up-regulated or down-regulated in X38. For mRNAs, 46 were up-regulated in X178 but down-regulated or unchanged in X38, 27 were down-regulated in X178 but up-regulated or unchanged in X38, and 77 were unchanged in X178 but either up-regulated or down-regulated in X38. To validate the high-throughput sequencing data, qRT-PCR was performed on selected lncRNAs and mRNAs. Linear regression analysis revealed a strong positive correlation (*R* ^2^ = 0.7758) between the qRT-PCR results and the sequencing data, confirming the reliability of the findings (**Figure 5**). Furthermore, based on lncRNA-mRNA interaction analysis, 25 Cd-tolerance-related lncRNAs and 128 target genes were identified (**Supplementary Table S8**). The distinct transcriptional profiles between the genotypes offer valuable insights into the mechanisms of Cd detoxification. The X178-specific upregulated lncRNAs may confer a tolerance advantage through three potential mechanisms: 1) Coordinating metal transporter dynamics for ion homeostasis, 2) Enhancing cellular antioxidant capacity via redox regulation, and 3) Fine-tuning stress signaling pathways. Conversely, the exclusive differential regulation of lncRNAs in X38 suggests that these transcripts may contribute to its Cd-sensitive phenotype, possibly by dysregulating metal sequestration pathways or impairing stress response coordination. This differential regulatory framework underscores genotype-specific molecular strategies in Cd response, with X178 activating protective lncRNA networks that remain inactive or suppressed in X38. The identified candidates provide a valuable foundation for further functional characterization of lncRNA-mediated Cd tolerance mechanisms in plants.

### 3.5 LncRNA target gene annotation and enrichment analysis

**Figure 6:**
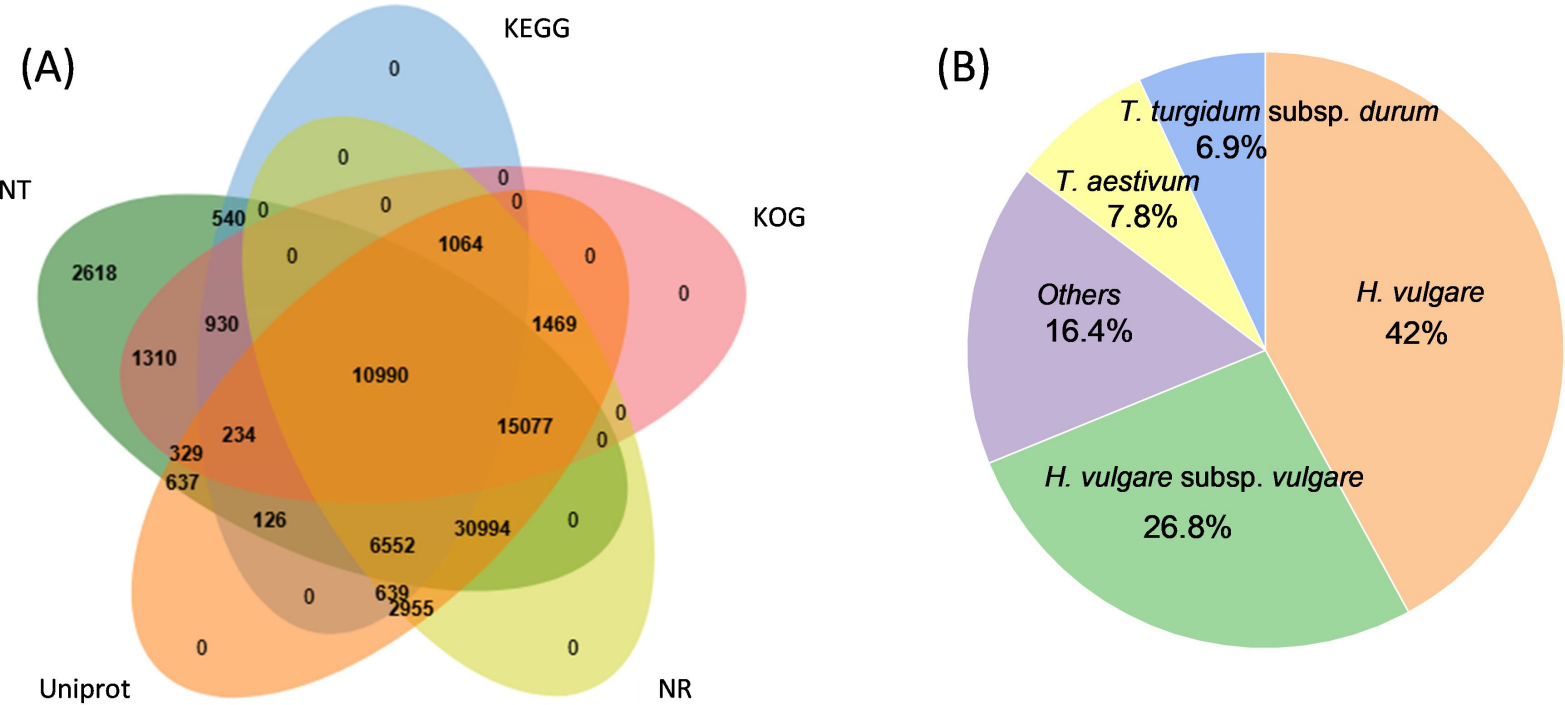
Annotation and function prediction of all mRNAs identified in four libraries. **(A)** Annotation of mRNAs by NR, NT, KEGG, KOG, Uniprot. Venn diagrams show number of mRNAs. **(B)** The species distribution of annotation results.

**Figure 7:**
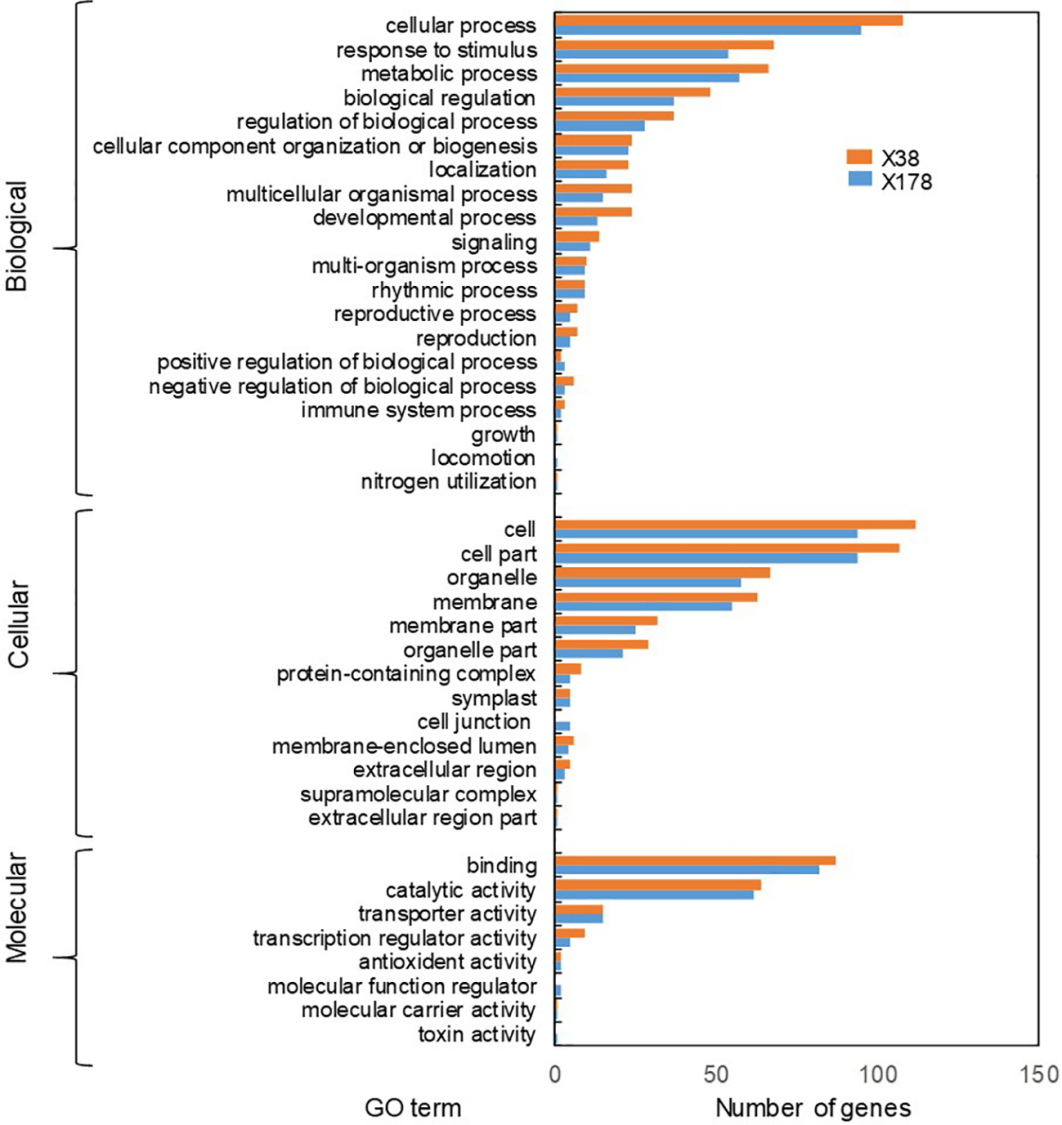
GO analysis of target genes of differentially expressed lncRNAs. GO classification of lncRNA target genes under Cd stress. The Y and X axes correspond to GO terms and the number of target genes (For interpretation of the references to colour in this figure legend, the reader is referred to the web version of this article).

**Figure 8:**
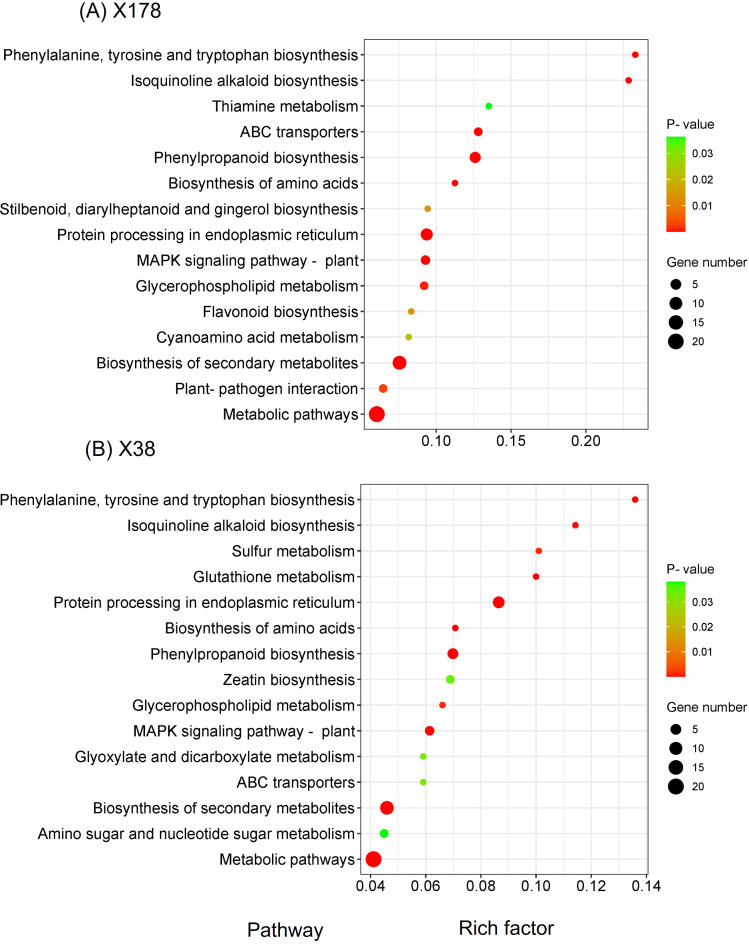
KEGG enrichment analysis of target genes of differentially expressed lncRNAs. KEGG pathway classification of target genes of lncRNAs under Cd stress in X178 **(A)** and X38 **(B)**, respectively. The Y axis corresponds to KEGG pathway, the X axis shows the enrichment ratio between the number of DEGs and all unigenes enriched in a particular pathway. The color of the dot represents p-value, and the size of the dot represents the number of DEGs mapped to the referent pathway.

The known and novel mRNAs were annotated using multiple databases, including NR, NT, GO, KOG, KEGG, UniProt, COG, Rfam, and TF. A total of 76,310 mRNAs, representing 96.05% of all mRNAs, were successfully annotated (**Supplementary Table S9**). Among these, 10,990 mRNAs were consistently annotated across all five databases (**Figure 6A**). The majority of the annotated mRNAs were mapped to *Hordeum vulgare* (42.04%), *Hordeum vulgare* subsp*. vulgare* (26.79%), *Triticum aestivum* (7.79%), and *Triticum* subsp. *durum* (6.95%) (**Figure 6B**). To gain deeper insights into the functional roles of the 128 lncRNA target genes, GO and KEGG enrichment analyses were performed. The GO annotation revealed that most genes in both genotypes were associated with cellular components such as cell and cell part, molecular functions including binding and catalytic activity, and biological processes such as cellular process, response to stimulus, and metabolic process (**Figure 7**). KEGG enrichment analysis highlighted several pathways that were significantly more enriched in the X178 genotype compared to X38 under Cd stress, suggesting potential mechanisms underlying enhanced Cd tolerance in X178 (**Figure 8**). These pathways included phenylalanine, tyrosine, and tryptophan biosynthesis, ABC transporters, and secondary metabolite biosynthesis, all of which are known to play roles in detoxification and stress responses. Additionally, X178 exhibited greater involvement in metabolic pathways and protein processing, which may contribute to maintaining cellular homeostasis under stress conditions. In contrast, X38, with fewer enriched pathways, appeared to be more susceptible to Cd stress, indicating that X178 has developed a more robust and generalized adaptive response to Cd exposure.

### 3.6 Key Cd-tolerance related lncRNAs and their targets

**Figure 9:**
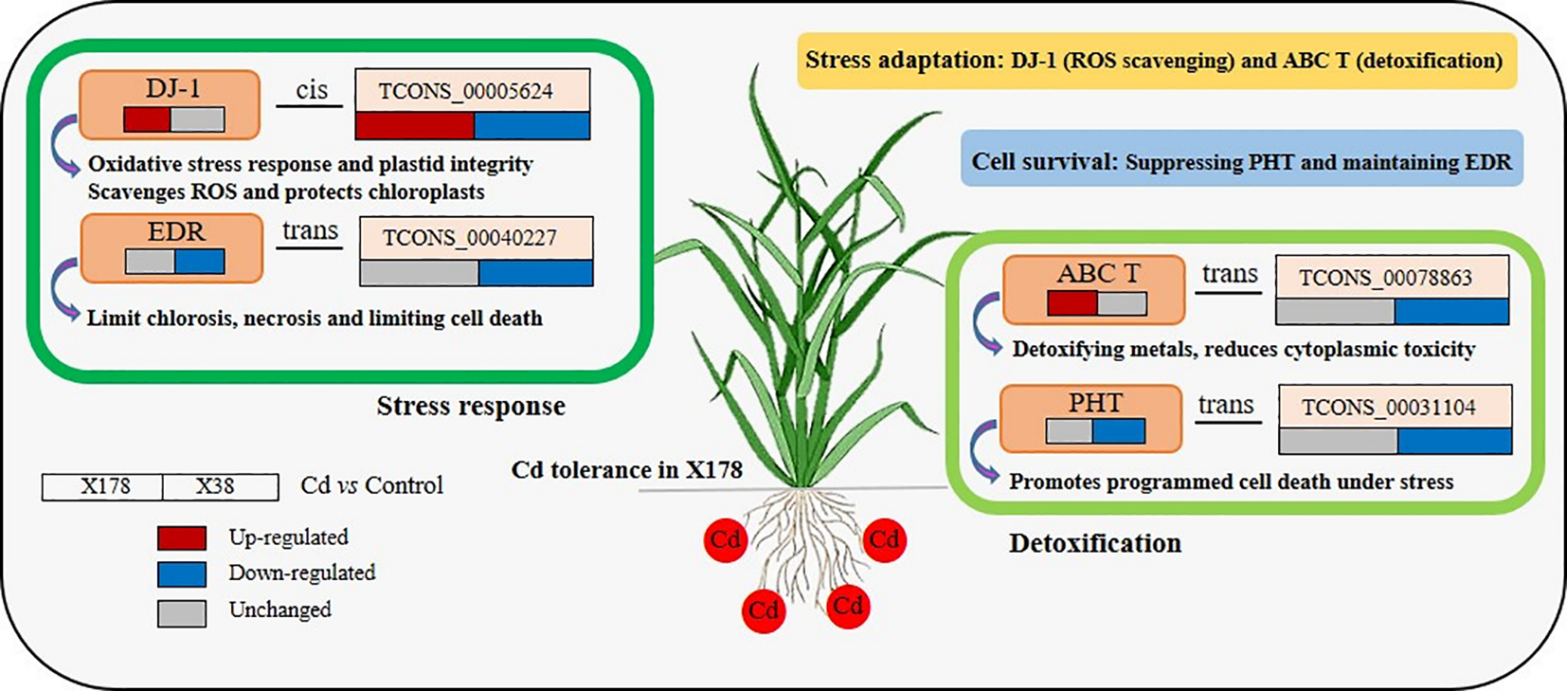
A hypothetical schematic illustration of the mechanism that underlies Cd tolerance and adaptation at the lncRNAs-mRNA level in X178. Within each genotype, the fold change (Cd vs control) is log_2_N, where changes in log_2_N ≥ 1 are up-regulated, 0 < |log_2_N| < 1 are unchanged and log_2_N ≤ –1 are down-regulated. For the box representing the expression, red, blue, and gray indicate up-regulated, down-regulated, and unchanged, respectively; the left and right represent X178 and X38. DJ-1, Protein DJ-1 homolog B; EDR, Protein enhanced disease resistance 2; ABC T, ABC transporter C family member 13; PHT, Putrescine hydroxycinnamoyltransferase 1.

We proposed 12 lncRNAs forming 18 lncRNA-mRNA pairs that are potentially critical for Cd tolerance (**Table 2**; **Figure 9**). Among these, lncRNA TCONS_00005624 was significantly up-regulated in X178 but down-regulated in X38, while its target gene TCONS_00005620 exhibited the same expression pattern, being up-regulated in X178 and down-regulated in X38. Another lncRNA, TCONS_00071028, was significantly down-regulated in X178 but remained unchanged in X38, with its target gene HORVU.MOREX.r2.6HG0471350.1 showing no change in X178 but up-regulation in X38. The remaining lncRNAs (TCONS_00035202, TCONS_00040227, TCONS_00073318, TCONS_00078863, TCONS_00029966, TCONS_00031104, TCONS_00086719, TCONS_00017042, TCONS_00047478, and TCONS_00007510) were unchanged in X178 but down-regulated in X38, with the exception of TCONS_00035202, which showed a different pattern. Among their target genes, 3 were up-regulated and 12 were unchanged in X178, while in X38, 8 were up-regulated, 5 were down-regulated, and 3 were unchanged. Based on functional annotation, the target genes of these lncRNAs were predicted to encode proteins such as protein DJ-1 homolog B, ABC transporter C family member 13, protein-enhanced disease resistance 2, and putrescine hydroxycinnamoyl transferase 1. These proteins are known to play essential roles in stress responses, metal detoxification, regulation of cell death, and plant growth and development, suggesting their potential involvement in Cd tolerance mechanisms. Additionally, several key genes, including TCONS_00013867 (transmembrane E3 ubiquitin-protein ligase), TCONS_00065833 (protein ENHANCED DISEASE RESISTANCE 2), TCONS_00086237 (ABC transporter C family member 13), and TCONS_00043280 (low affinity sulfate transporter 3), were validated by qRT-PCR (**Figure 5**), further supporting their involvement in genotype-specific tolerance in X178.

**Table 2:** Cd tolerant related lncRNAs and their target mRNAs in response to Cd.

LncRNA ID	Fold change (Cd vs control)		Target mRNA	Fold change (Cd vs control)		Annotation
	X178	X38		X178	X38	
TCONS_00005624	21.28	-4.23	TCONS_00005620	1.08	-0.48	Protein DJ-1 homolog B
TCONS_00071028	-1.08	-0.64	HORVU.MOREX.r2.6HG0471350.1	0.93	1.18	Cinnamoyl-CoA reductase 1
TCONS_00035202	0.96	1.20	TCONS_00013867	1.13	0.71	Transmembrane E3 ubiquitin-protein ligase
TCONS_00040227	-0.78	-1.27	TCONS_00065833	-0.54	-1.20	Protein ENHANCED DISEASE RESISTANCE 2
TCONS_00073318	-0.94	-1.23	HORVU.MOREX.r2.7HG0544860.1	-0.89	-1.15	HNHc domain-containing protein
TCONS_00078863	-0.91	-1.22	TCONS_00086237	1.03	0.76	ABC transporter C family member 13
TCONS_00029966	-0.97	-1.40	HORVU.MOREX.r2.1HG0013370.1	0.98	1.69	Peroxidase 2
TCONS_00029966	-0.97	-1.40	HORVU.MOREX.r2.4HG0320560.1	0.80	1.42	Probable glucuronosyltransferase Os03g0287800
TCONS_00029966	-0.97	-1.40	HORVU.MOREX.r2.4HG0340350.1	0.67	1.35	2-oxoglutarate-dependent dioxygenase 11
TCONS_00029966	-0.97	-1.40	HORVU.MOREX.r2.5HG0364740.1	0.60	1.37	Uncharacterized protein
TCONS_00029966	-0.97	-1.40	TCONS_00024166	0.48	1.12	Polygalacturonase QRT3
TCONS_00029966	-0.97	-1.40	TCONS_00043280	0.88	1.04	Low affinity sulfate transporter 3
TCONS_00031104	-0.63	-1.16	HORVU.MOREX.r2.7HG0620250.1	-0.77	-1.02	Putrescine hydroxycinnamoyltransferase 1
TCONS_00086719	-0.77	-1.05	TCONS_00016866	0.78	1.48	Galacturonokinase;
TCONS_00017042	-0.75	-1.16	HORVU.MOREX.r2.7HG0605000.1	0.93	1.52	Luminal-binding protein 3
TCONS_00047478	-0.92	-1.08	HORVU.MOREX.r2.2HG0112600.1	-0.91	-1.06	Mixed-linked glucan synthase 4
TCONS_00007510	-0.61	-1.49	TCONS_00022788	-0.57	-1.19	Alpha-glucan phosphorylase 1
TCONS_00007510	-0.61	-1.49	TCONS_00075108	1.01	0.99	Oligopeptide transporter 7

Fold change (Cd vs control) is log_2_N, log_2_N ≥ 1.0 are up-regulated, between 0 < |log_2_N| < 1.0 are unchanged and log_2_N ≤ − 1.0 are down-regulated.

## 4 Discussion

The detrimental impact of Cd stress on crop yield and quality is well-documented, as it disrupts numerous physiological and biochemical processes in plants. Recent advancements in high-throughput RNA-seq technologies have generated vast amounts of transcriptomic data, shedding light on the complexity of eukaryotic transcriptomes and the pivotal role of lncRNAs in regulating gene expression. These discoveries have provided novel insights into plant stress responses. Previous study identifies at least a thousand lncRNAs that have been found in several species under biotic and abiotic stress, highlighting their potential in stress response pathways (Chen et al., 2019; Zheng W, et al., 2023; Zou et al., 2024). LncRNAs have been implicated in regulating multiple physiological pathways, including responses to Cd stress. However, despite their broad functional roles, the precise regulatory mechanisms of lncRNAs remain poorly understood. To date, only a limited number of studies have conducted genome-wide characterizations of lncRNAs involved in plant responses to Cd stress, with notable examples in *Oryza sativa* (Chen et al., 2018; Feng et al., 2016a), *Hordeum vulgare* (Zhou et al., 2023), *Betula platyphylla* (Wen et al., 2020), and *Populus tomentosa* (Quan et al., 2021). Experimental evidence has demonstrated that specific lncRNAs play critical roles in modulating Cd stress responses. For instance, Chen et al. (2018) reported that lncRNAs may regulate genes involved in cysteine-rich peptide metabolism in cis, as well as secondary metabolites and photosynthesis in trans, thereby triggering a cascade of physiological and biochemical responses to elevated Cd levels. Furthermore, heterologous overexpression of *PtoMYB73* and *PtoMYB27* in *Arabidopsis* revealed their roles in enhancing Cd tolerance, photosynthetic efficiency, and leaf growth, while also elucidating potential mechanisms of interaction with abiotic stressors (Quan et al., 2021).

In our previous research, we identified Tibetan hull-less barley genotypes X178 and X38 as contrasting models for Cd tolerance, with X178 exhibiting greater Cd tolerance and X38 showing heightened susceptibility to Cd stress (Foysal et al., 2024). This study also investigated the differences in plant physiological and biochemical characteristics (growth and morphological parameters, photosynthetic and chlorophyll-related traits, oxidative stress and antioxidant enzyme activities, Cd uptake and translocation), as well as mineral nutritional responses to Cd stress. These genotypes serve as valuable germplasm resources for unraveling the molecular mechanisms underlying Cd tolerance in crops and identifying associated genes. In this study, we employed next-generation RNA sequencing to compare Cd-tolerant and Cd-sensitive Tibetan wild barley genotypes, aiming to uncover Cd-related lncRNAs and genes under Cd stress. Based on our screening standards, we found 8299 novel lncRNAs and 15791 novel mRNAs in barley roots. Notably, Cd stress significantly altered the expression profiles of lncRNAs in both genotypes, with 26 differentially expressed lncRNAs identified as potential key regulators of Cd tolerance in X178. LncRNAs and small RNAs (sRNAs) are critical transcriptional regulators that can interact with each other, with lncRNAs often serving as precursors for sRNAs. For example, Chen et al. (2016) demonstrated that in poplar under nitrogen deficiency, nine intergenic lncRNAs acted as precursors for 11 known miRNAs. Similarly, in *Brassica napus* under Cd stress, four lncRNAs were identified as precursors for miR824, miR167d, miR156d, and miR156e (Feng et al., 2016b). In our study, computational predictions revealed that 25 lncRNAs may function as precursors for miRNAs in related species, highlighting the importance of lncRNAs in miRNA regulation during Cd stress.

Although lncRNAs do not encode proteins, they produce functional RNA molecules that regulate gene expression through diverse mechanisms, including cis and trans transcriptional regulation, post-transcriptional modulation, and chromatin structure remodeling. Studies have shown that long intronic and antisense noncoding RNAs can promote the accumulation of histone 3 lysine 27 trimethylation (H3K27me3), leading to epigenetic silencing (Csorba et al., 2014; Heo and Sung, 2010). For instance, antisense lncRNAs have been identified in poplar under nitrogen deficiency (Chen et al., 2016) and in alfalfa under salt stress (Postnikova and Nemchinov, 2015), where they form double-stranded RNA duplexes with sense mRNAs, thereby influencing gene expression on the opposing strand. In the Cd-tolerant genotype X178, we identified 12 lncRNAs associated with Cd stress. Among these, one lncRNA was upregulated, one was downregulated, and ten exhibited no significant change in expression. Interestingly, their target genes showed varied expression patterns, with four upregulated and eight unchanged in X178 under Cd stress. The regulatory dynamics between lncRNAs and their putative target genes are complex and require further experimental validation. Unlike the well-characterized inverse relationship between miRNAs and their target genes, the interaction patterns of lncRNAs are less understood. In our study, we predicted that the target genes of lncRNAs include protein DJ-1 homolog B, which is crucial for stress responses. The DJ-1 superfamily, widely conserved across all kingdoms of life, plays a significant role in stress responses. Recent findings by Rathore et al. (2024) demonstrate that *OsDJ-1C* overexpression lines exhibit reduced yield penalties under drought and salinity stress compared to wild-type plants, accompanied by enhanced photosynthetic efficiency and improved root architecture. The observed root system expansion in these transgenic lines potentially results from adaptive mechanisms involving root development-associated genes, including ROS scavenging systems (Bhaskarla et al., 2020), which may activate antioxidant defense pathways to confer stress tolerance (Zhang et al., 2014).

Additionally, target genes of lncRNAs were predicted to include ABC transporter C family member 13 (ABC T), protein-enhanced disease resistance 2 (EDR), and putrescine hydroxycinnamoyl transferase 1 (PHT). ABC transporters are known to facilitate metal detoxification by transporting metals across membranes, often sequestering them in vacuoles or expelling them from cells. As demonstrated by Kou et al. (2024), ABC transporters constitute critical components in plant stress responses by actively removing heavy metals, toxic ions, and stress-related metabolites from cells, thereby mitigating stress-induced cellular damage. This aligns with previous findings by Fu et al. (2019) showing that OsABCG36, a G-type ABC transporter, confers Cd tolerance in rice. Additionally, Xiao et al. (2024) recently expanded our understanding by elucidating ABC transporters’ pivotal roles in selenium homeostasis and stress adaptation in soybean. Another important gene, EDR2, is strongly linked to stress response pathways. Neubauer et al. (2020) provided mechanistic insights, demonstrating that the *Arabidopsis* EDR1 kinase regulates programmed cell death by modulating the EDS1-PAD4 complex. Furthermore, PHT is involved in the regulation of cell death, with Fang et al. (2022) reporting that OsPHT3 and OsPHT4 positively regulate cell death in rice. While we observed limited expression patterns of these genes in the Cd-tolerant genotype X178, their precise roles remain unclear and warrant further investigation. Taken together, we propose that the key lncRNA-mRNA interactions related to Cd tolerance might modulate the expression of proteins such as DJ-1 homolog B and EDR2, shedding light on their potential involvement in stress responses. While plant hormones are known to function as signaling molecules that coordinate complex response mechanisms to Cd stress (Küçükrecep et al., 2024), the target genes identified in this study were not directly linked to plant hormones. Future research could explore the potential role of plant hormone-related transcriptional activation.

The revealed lncRNAs may be applied as targets for genetic engineering or as molecular markers for breeding initiatives to create Cd-tolerant barley cultivars. Furthermore, lncRNA profiles may aid in the selection and identification of barley lines resistant to Cd, accelerating the creation of vigorous cultivars. This study provides valuable insights into the role of lncRNAs in Cd tolerance in barley. However, it has several limitations: the study focused on a single concentration of treatment and a single stress condition (Cd exposure); the functional validation of the identified lncRNAs was limited, leaving their precise mechanisms of action unclear; and the responses of lncRNAs under different levels of Cd stress and combined stresses remain unexplored. Future research should include functional characterization of lncRNAs using CRISPR/Cas9 or overexpression techniques, investigate their roles under varying Cd levels and durations, and assess their effects under combined stress conditions. Moreover, confirming these findings in field trials would be essential for practical applications.

## 5 Conclusion

This study established comprehensive lncRNA-mRNA regulatory networks underlying Cd tolerance in Tibetan hull-less barley, revealing critical interactions between 26 differentially expressed lncRNAs and 150 mRNAs in response to Cd stress. By integrating cis- and trans-regulatory mechanisms, these networks highlighted the role of lncRNAs in modulating genes associated with detoxification pathways, ABC transporters, and secondary metabolite biosynthesis. Notably, 12 lncRNAs forming 18 functional pairs were identified as key regulators of stress-responsive proteins, including DJ-1, EDR, PHT, and ABC transporter. These findings not only elucidate the molecular basis of lncRNA-mediated Cd tolerance but also provide potential targets for breeding or engineering crops resilient to heavy metal stress. This advances our understanding of lncRNAs as pivotal regulators in plant abiotic stress responses. Given the conserved nature of heavy metal response mechanisms across plant species, the identified regulatory networks in barley may reveal universal strategies for improving metal tolerance in agriculturally important crops. This work establishes a conceptual framework for developing Cd-resistant cultivars across diverse plant species, offering potential solutions for cultivation in contaminated environments. Future investigations should prioritize functional validation of candidate lncRNAs through molecular characterization, while exploring practical applications through lncRNA-based genetic engineering approaches and marker-assisted selection programs under Cd stress conditions to confirm these regulatory relationships.

## Data Availability

The original contributions presented in the study are included in the article/**Supplementary Material**. Further inquiries can be directed to the corresponding author/s.
