# Supplementary material for: Comparative transcriptome analysis reveals key long noncoding RNAs for cadmium tolerance in Tibetan hull-less barley
Source: Front Plant Sci. 2025 May 22;16:1572490. doi: 10.3389/fpls.2025.1572490 (PMC12138524; doi:10.3389/fpls.2025.1572490)
Supplement: Supplementary file 1 [file DataSheet1.zip › Supplementary Figures.docx]

**Figure S1.** Phenotypical observation of X178 and X38 under control and 20 µmol L^–1^ Cd stress after 10 days of treatment.

**Figure S2.** Growth parameters of X178 and X38 under control and 20 µmol L^–1^ Cd stress after 15 days of treatment. (A-F) shoot height, root length, shoot fresh weight, shoot dry weight, root fresh weight, root dry weight. FW = fresh weight, DW = dry weight. One-way ANOVA was used, and multiple comparisons were made using Duncan’s test. Different letters indicate significant differences at *P* < 0.05.
