# Supplementary material for: Comparative transcriptome analysis reveals key long noncoding RNAs for cadmium tolerance in Tibetan hull-less barley
Source: Front Plant Sci. 2025 May 22;16:1572490. doi: 10.3389/fpls.2025.1572490 (PMC12138524; doi:10.3389/fpls.2025.1572490)
Supplement: Supplementary file 1 [file DataSheet1.zip › Table S1. List of primers used for qRT-PCR of lncRNA and mRNAs.docx]

Supplementary Table S1. List of primers used for qRT-PCR of lncRNA and mRNAs

| **Transcript ID** | **Transcript type** | **Primer** | **Sequence** |
| --- | --- | --- | --- |
| TCONS_00073318 | lncRNA | Forward | ACCAAGGGGCATGTCTTCAG |
|  |  | Reverse | ACCATGCTGGTGTAGTGGAC |
| TCONS_00078863 | lncRNA | Forward | TCTGGTCACCCGGAGGTTTA |
|  |  | Reverse | ACTAATCCAGCAAGGCCACC |
| TCONS_00031104 | lncRNA | Forward | ATCAGCAACCTTGCCCATGA |
|  |  | Reverse | ATATAGAAAGCCGGCGGGGA |
| TCONS_00086719 | lncRNA | Forward | AACCGTGGACCGGATTAAAA |
|  |  | Reverse | GACGCAAACCGATGCAAACA |
| TCONS_00017042 | lncRNA | Forward | ACGATCGGGGTTGTTATGGG |
|  |  | Reverse | CGGGTTCATGACGCCCTATG |
| TCONS_00007510 | lncRNA | Forward | CGTCATGAACCCGAGGAGTA |
|  |  | Reverse | GAAGCGTACGAGTGCCAAAC |
| TCONS_00013867 | mRNAs | Forward | TTGTAAAGGTGGCGACCCAA |
|  |  | Reverse | TTATGAGCCGGCCTGTCAAG |
| TCONS_00065833 | mRNAs | Forward | GTTACGCAACGACGACACTC |
|  |  | Reverse | CGGTTCATCCTAGACGGCAA |
| TCONS_00086237 | mRNAs | Forward | GGTGGAACTGCGTGTTGTTA |
|  |  | Reverse | AGGAATGGAGGAAGTACTTAGGA |
| TCONS_00043280 | mRNAs | Forward | CGGGCAACTTCTTCATCGGA |
|  |  | Reverse | GTCATGTCAGTGTTGCGGTC |
| *GAPDH* | Reference gene | Forward | AAGCATGAAGATACAGGGAGTGTG |
|  |  | Reverse | AAATTTATTCTCGGAAGAGGTTGTACA |
